# Groove area involvement predicts post-ERCP pancreatitis after 8-mm fully-covered metal stent placement in resectable pancreatic cancer

**DOI:** 10.1055/a-2803-4865

**Published:** 2026-02-26

**Authors:** Shinya Kawaguchi, Eiji Nakatani, Tatsunori Satoh, Shodai Takeda, Yuichi Masui, Shinya Endo, Hideyuki Kanemoto

**Affiliations:** 1 Gastroenterology, Shizuoka General Hospital, Shizuoka, Japan; 2 Department of Biostatistics and Health Data Science, Graduate School of Medical Science, Nagoya City University, Nagoya, Japan; 3 Surgery, Shizuoka General Hospital, Shizuoka, Japan

**Keywords:** Pancreatobiliary (ERCP/PTCD), Strictures, ERC topics

## Abstract

**Background and study aims:**

Fully covered self-expandable metal stents (FCSEMSs) provide durable preoperative biliary drainage in pancreatic cancer but may increase risk of post-endoscopic retrograde cholangiopancreatography (ERCP) pancreatitis (PEP). We evaluated whether groove involvement was an independent anatomical PEP risk factor and compared PEP incidence after 8-mm FCSEMS and plastic stent (PS) placement using propensity-score–based inverse probability of treatment weighting (IPTW).

**Patients and methods:**

Sixty-two consecutive patients with resectable or borderline resectable pancreatic cancer and distal biliary strictures with naïve papillae underwent ERCPs between February 2015 and August 2024. An 8-mm FCSEMS or PS (7–11.5F) was placed. Independent PEP predictors were identified using multivariable Firth logistic regression. PEP incidence was compared between stent types after IPTW adjustment for age, sex, clinical stage, groove involvement, main pancreatic duct diameter, and prophylactic pancreatic-stent placement.

**Results:**

Mean age was 73.3 ± 8.2 years (62.9% male). Groove-area extension was present in 21.0% of tumors. PEP occurred in six patients (9.7%), all after FCSEMS placement. Groove involvement independently predicted PEP (adjusted odds ratio, 14.7; 95% confidence interval, 2.26–95.9; *P* = 0.005). After IPTW, the weighted PEP rate remained higher with FCSEMS than PS (13.4% vs 0%; *P* = 0.011).

**Conclusions:**

Groove-area tumor extension is an independent imaging-detectable PEP risk factor. Even after baseline difference adjustment, 8-mm FCSEMS placement was associated with a higher pancreatitis risk than PS placement. Pre-procedural groove involvement identification may guide stent selection and support selective prophylactic pancreatic stenting. However, further confirmation through larger prospective studies is required.

## Introduction

Obstructive jaundice in patients with resectable or borderline-resectable pancreatic cancer often requires preoperative biliary drainage, particularly during neoadjuvant therapy. The European Society of Gastrointestinal Endoscopy (ESGE) 1 recommend 10-mm self-expandable metal stents (SEMSs) rather than plastic stents (PSs) because SEMSs maintain patency longer and lower risk of recurrent biliary obstruction (RBO).

However, large-caliber SEMSs have been linked to a high incidence of post-endoscopic retrograde cholangiopancreatography (ERCP) pancreatitis (PEP) 2 3 4 . To mitigate this problem, investigators have explored smaller-diameter, fully-covered SEMSs (FCSEMSs) 5 6 . A recent multicenter prospective study that used 7-mm FCSEMSs did not demonstrate a clear reduction in PEP rates and offered no direct comparison with PSs 7 .

At our institution, we observed that patients whose tumors extended into the “groove area,” (the narrow space bounded by the pancreatic head, duodenum, and common bile duct) appeared to develop PEP more frequently after placement of 8-mm FCSEMSs. This finding, based on an unpublished prospective series, suggests that groove involvement might be an underrecognized anatomical risk factor detectable on routine cross-sectional imaging before the procedure.

Previous studies have primarily focused on technical and pharmacological determinants of PEP. Only a few studies have examined anatomical markers available before endoscopic intervention. Song et al. proposed stent selection criteria based on the obstruction type in malignant biliary strictures 8 , and Zeng et al. summarized prophylactic measures to prevent PEP 9 . Umemura et al. reported that main pancreatic duct (MPD) dilatation is associated with a significantly lower risk of PEP after transpapillary stenting, suggesting that pre-procedure pancreatic duct morphology may serve as a protective anatomical factor 10 . However, none of these reports have specifically addressed the relevance of groove-area extensions.

Therefore, the present retrospective study aimed to identify independent risk factors for PEP, with particular attention to groove involvement, and compare incidence of PEP between patients who had 8-mm FCSEMSs placed and those who had PSs placed during preoperative biliary drainage.

## Patients and methods

### Study design and population

This single-center, retrospective, observational study was approved by the Institutional Review Board of Shizuoka General Hospital (SGHIRB #2024081). Patients who underwent biliary stent placement via ERCP for distal biliary strictures associated with resectable or borderline-resectable pancreatic cancer between February 2015 and August 2024 were included. Inclusion criteria were as follows: 1) patients with naïve papillae; and 2) each patient received either an 8-mm FCSEMS or a PS (7–11.5F). Unresectable cases were excluded because, despite PEP being a short-term adverse event (AE), biliary drainage strategies, stent selection, and procedure intent differ substantially from the preoperative setting, introducing clinical heterogeneity and confounding by indication.

### ERCP procedures

All ERCP procedures were performed under intravenous sedation with continuous vital-sign monitoring. In accordance with ESGE recommendations, a single rectal dose of diclofenac (25–50 mg) was administered immediately before the procedure in all patients, except those with contraindications. However, diclofenac was not used in cases performed prior to April 2018. This reflects a temporal change in institutional practice rather than patient-specific risk stratification. Protease inhibitors and aggressive hydration with lactated Ringer’s solution were not routinely administered. Endoscopic sphincterotomy (EST) was performed before placement of any stent ≥ 8.5F, and especially before FCSEMS deployment, to minimize insertion resistance. When the guidewire unintentionally cannulated the pancreatic duct, or when biliary cannulation proved difficult, a prophylactic endoscopic pancreatic stent (EPS) was inserted at the discretion of the endoscopist. EPS was not routinely placed for difficult biliary cannulation alone in absent pancreatic duct cannulation. The stent selection criteria were as follows. Before June 2019, when neoadjuvant chemotherapy (NAC) was introduced at our institution in accordance with the Japanese Clinical Practice Guidelines for Pancreatic Cancer 2019 11 , PSs were used in all cases. Since then, FCSEMSs generally have been selected for patients in whom NAC was anticipated at the time of ERCP as a waiting period before surgery was expected. The final decision regarding stent type and calibration, including PSs, was made at the discretion of the endoscopist performing the procedure. All procedures were supervised by pancreaticobiliary endoscopists with experience in > 400 ERCP procedures.

### Groove-area tumors

A groove-area tumor was defined as a lesion involving the anatomical space bounded by the pancreatic head, duodenum, and common bile duct, as identified on contrast-enhanced computed tomography (CT). All CT images were interpreted by board-certified radiologists as part of routine clinical practice and groove involvement was documented in the original radiological reports prior to ERCP.

### Adverse events

All AEs that occurred within 30 days of ERCP were recorded. Events specific to transpapillary stenting were categorized according to the Tokyo Criteria 2014 12 , and severity was graded according to the AGREE classification, a structured system based on the Clavien-Dindo surgical grading scale 13 .

### PEP

PEP was diagnosed in accordance with Cotton et al.’s criteria 14 , when new or worsening abdominal pain typical of pancreatitis was accompanied by a serum amylase level more than three times the upper limit of normal within 24 hours after ERCP and the patient required at least a two-night hospital stay or an extension of the current admission by two nights or more. Severity was classified as mild (hospital stay 2–3 days), moderate (4–10 days), or severe (> 10 days or presence of local or systemic complications), which correspond to those of the American Society for Gastrointestinal Endoscopy Lexicon criteria 15 .

### RBO

RBO was defined as recurrence of jaundice or cholangitis that necessitated repeat endoscopic or percutaneous intervention before surgical resection, or before a definitive decision against surgery.

### Statistical analyses

Continuous variables are expressed as mean ± standard deviations. Clinical success, AEs, and PEP incidence were compared between the groups. Risk factors for PEP were assessed through univariate and multivariate logistic regression analyses using Firth’s penalized likelihood to mitigate small-sample bias. Odds ratios (ORs) and 95% confidence intervals (CIs) were calculated. Baseline differences between the FCSEMS and PS groups were assessed using inverse probability of treatment weighting (IPTW) based on propensity scores derived from age, sex, clinical stage, tumor location (groove vs. non-groove), MPD diameter, and EPS placement. Propensity score matching was not performed because the limited number of groove-involved cases could have resulted in substantial case loss and unstable estimates. Covariate balance was considered adequate when the standardized mean difference (SMD) was < 0.20. Statistical significance was defined as a two-sided *P* < 0.05. All analyses were performed using EZR (Saitama Medical Center, Jichi Medical University), a graphical interface for R equipped with additional biostatistical functions.

## Results

### Baseline characteristics

Sixty-two patients met the inclusion criteria. Mean age was 73.3 ± 8.2 years and 39 (62.9%) were male. Tumor resectability was classified as resectable in 54 patients, borderline resectable with portal-vein involvement (BR-PV) in five, and borderline resectable with arterial involvement (BR-A) in three. Stage IIA disease accounted for 48 cases and stage IIB disease accounted for 14. Groove involvement was observed in 13 patients (21.0%). Median tumor diameter was 25.0 mm (interquartile range [IQR], 18.3–30.0 mm). Detailed baseline data are summarized in Table 1 .

**Table:** **Table 1** Baseline characteristics of study patients (n = 62).

**Variable**	**Category/unit**	**Value**
Age	Years (mean ± SD)	73.3 ± 8.2
Sex	Male	39 (62.9%)
Resectability classification	Resectable	54 (87.1%)
	Borderline resectable (BR-PV)	5 (8.1%)
	Borderline resectable (BR-A)	3 (4.8%)
UICC stage (8th edition)	IIA	48 (77.4%)
	IIB	14 (22.6%)
Location of pancreatic tumor	Head	49 (79.0%)
	Groove	13 (21.0%)
Maximum tumor size	mm (median, IQR)	25 (18.3–30.0)
Pancreatic duct diameter	mm (median, IQR)	4 (2.1–7.0)
Tumor involvement of the cystic duct	Presence	1 (1.6%)
History of cholecystectomy	Presence	2 (3.2%)
Data are presented as mean ± standard deviation for continuous variables and as numbers (%) for categorical variables. Pancreatic duct diameter is expressed as medians and interquartile ranges. BR-A, borderline resectable with arterial involvement; BR-PV, borderline resectable with portal vein involvement; IQR, interquartile range; SD, standard deviation; UICC, Union for International Cancer Control.		

### Procedure data

An 8-mm FCSEMS was placed in 45 patients (72.6%) and a PS in 17 (27.4%). EST was performed in 50 patients (80.6%) and prophylactic EPS in 12 (19.4%). Median cannulation time was 8 minutes (interquartile range [IQR] 3–22), and median procedure time was 24 minutes (IQR 18–39). Diclofenac was used for prevention of PEP in 38 patients (61.3%) ( Table 2 ).

**Table:** **Table 2** ERCP-related procedure information (n = 62).

**Variable**	**Category**	**Value**
Cannulation time	min (median, IQR)	8 (3–22)
Procedure time	min (median, IQR)	24 (18–39)
EST	Performed	50 (80.6%)
Type of stent	FCSEMS (8 mm)	45 (72.6%)
	PS (including 7F, 8.5F, 11.5F) ^*^	17 (27.4%)
	PS (7F)	13 (21.0%)
	PS (8.5F)	1 (1.6%)
	PS (11.5F)	3 (4.8%)
EPS placement	FCSEMS and PS ^†^	12 (19.4%)
	FCSEMS	5 (8.1%)
	PS	7 (11.3%)
Drugs for prevention of PEP	Diclofenac	38 (61.3%)
EST, endoscopic sphincterotomy; EPS, endoscopic pancreatic duct stent; FCSEMS, fully-covered self-expandable metal stent; IQR, interquartile range; PEP, post-endoscopic retrograde cholangiopancreatography pancreatitis; PS, plastic stent. ^*^ Data are presented as numbers (%). “PS” includes 7F, 8.5F, and 11.5F plastic stents. ^†^ EPS placement includes patients who underwent pancreatic duct stenting with either or both types of stents. Cannulation times and procedure times are expressed as median and interquartile ranges.		

### Clinical outcomes and AEs

Technical and clinical biliary drainage success was achieved in 60 of 62 patients (96.8%). Two patients in the FCSEMS group required upfront surgery because of persistent jaundice despite patent stents. RBO occurred in the two PS recipients (3.2%) and was managed endoscopically without subsequent migration.

Fourteen patients (22.6%) experienced at least one AE. PEP developed in six patients (9.7%): all six had received FCSEMSs, yielding unadjusted PEP rates of 13.3 % for FCSEMSs and 0% for PSs. Acute cholecystitis occurred in five patients (8.1%), cholangitis in one (1.6%), liver abscess in one (1.6%), and post-EST bleeding in one (1.6%) ( Table 3 ). All AEs were mild and resolved with standard therapy.

**Table:** **Table 3** Clinical outcomes and adverse events (n = 62).

**Variable**	**Category**	**n (%)**
**Clinical outcome**		
Clinical success	Achieved	60 (96.8%)
Recurrent biliary obstruction	Due to migration	0 (0.0%)
	Due to stent obstruction	2 (3.2%)
Number of re-interventions		2 (3.2%)
**Adverse events**		
Acute pancreatitis (PEP)	Occurred	6 (9.7%)
	Mild	6 (9.7%)
	Moderate	0 (0.0%)
Acute cholecystitis	Occurred	5 (8.1%)
	Mild	3 (4.8%)
	Moderate	2 (3.2%)
Cholangitis	Occurred	1 (1.6%)
	Mild	1 (1.6%)
	Moderate	0 (0.0%)
Liver abscess	Occurred	1 (1.6%)
Post-EST bleeding	Occurred	1 (1.6%)
**Intervention for AEs**		
ENPD	Occurred	1 (1.6%)
PTGBD	Occurred	1 (1.6%)
ENBD	Occurred	2 (3.2%)
Endoscopic hemostasis	Occurred	1 (1.6%)
EST, endoscopic sphincterotomy; ENBD, endoscopic nasobiliary drainage; ENPD, endoscopic nasopancreatic drainage; PEP, post-endoscopic retrograde cholangiopancreatography pancreatitis; PTGBD, percutaneous transhepatic gallbladder drainage. Data are presented as numbers (%), unless otherwise specified. Adverse events were graded as mild or moderate.		

**Fig. 1:**
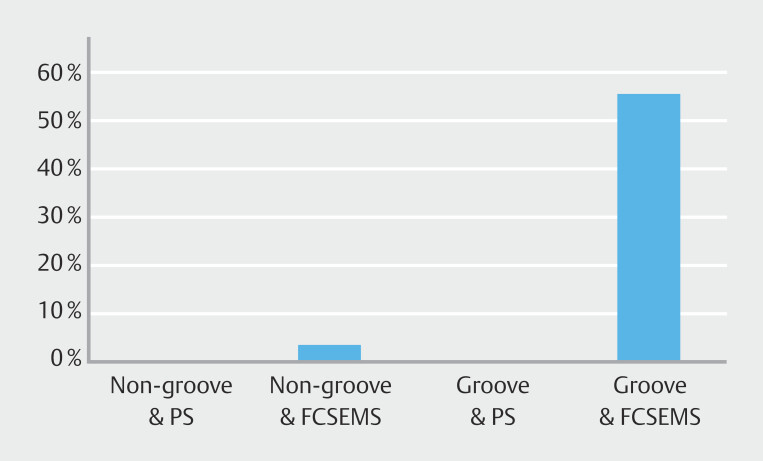
PEP incidence by PS and FCSEMS placement in groove involvement and non-groove involvement cases.

Among FCSEMS recipients, incidence of PEP differed markedly by tumor location: 55.6% in groove involvement versus 2.8% in non-groove tumors ( Fig. 1 ).

### Risk factor analysis for PEP

In univariate analysis, groove involvement was strongly associated with PEP (OR 30.0; 95% CI 3.09–291.4; *P* = 0.003). Multivariate Firth-adjusted logistic regression confirmed groove involvement as the sole independent predictor (adjusted OR 14.7; 95% CI 2.26–95.9; *P* = 0.005). Neither MPD diameter < 3 mm, EPS placement, nor use of diclofenac influenced risk of PEP ( Table 4 ).

**Table:** **Table 4** Results of univariable and multivariable logistic regression analyses for PEP.

**Variable**	**Category or unit**	**PEP**		**Odds ratio**	**95% CI**	***P* value **	**Odds ratio**	**95% CI**	***P* value **
		**Absent**	**Occurred**						
		**N = 56**	**N = 6**						
Sex	Female	22	1	1		0.299			
	Male	34	5	3.23	0.35–29.56				
Age, mean ± SD	1 year	73.21 (8.54)	74.33 (5.28)	1.02	0.91–1.13	0.751			
Resectability	R	48	6	1		0.972			
	BR-A	3	0	1.07	0.03–36.1				
	BR-PV	5	0	0.68	0.03–17.98				
UICC stage	IIA	44	4	1		0.512			
	IIB	12	2	1.83	0.30–11.24				
Location	Head	48	1	1		0.003	1		0.005
	Groove	8	5	30	3.09–291.38		14.7	2.26–95.89	
Tumor size, mean ± SD	1 mm	24.84 (7.81)	24.50 (8.69)		0.46–13.71	0.921			
MPD < 3 mm	No	40	3	1		0.291	1		0.697
	Yes	16	3	2.5	0.46–13.71		1.44	0.23–8.92	
T. Bil, mean ± SD	1 mg/dL	10.34 (7.67)	14.57 (5.08)	1.08	0.96–1.20	0.199			
Type of stent	PS	17	0	1		0.255			
	FCSEMS	39	6	5.76	0.28–117.04				
EPS placement	No	44	6	1		0.406	1		0.649
	Yes	12	0	0.27	0.01–5.82		0.47	0.02–12.10	
EST	No	12	0	1		0.406			
	Yes	44	6	3.65	0.17–77.65				
Diclofenac use	No	21	3	1		0.669			
	Yes	35	3	0.632	0.14–2.88				
BR, borderline resectable; CI, confidence interval; EPS, endoscopic pancreatic duct stent; EST, endoscopic sphincterotomy; FCSEMS, fully covered self-expandable metal stent; MPD, main pancreatic duct; OR, odds ratio; PEP, post-endoscopic retrograde cholangiopancreatography pancreatitis; PS, plastic stent; SD, standard deviation; T. Bil, total bilirubin; UICC, Union for International Cancer Control. Odds ratios and 95% CIs were estimated using Firth’s penalized likelihood logistic regression to reduce small-sample bias. Multivariable analysis included variables with P < 0.10 in univariable analysis or deemed clinically relevant.									

### Comparison between FCSEMSs and PSs after covariate adjustment

Because no PEP occurred in the PS group, conventional regression analysis was not feasible. Baseline characteristics before weighting are summarized in **Supplementary Table 1** . After IPTW, the weighted cohorts were well balanced (all SMDs < 0.20) ( Table 5 ). In the weighted analysis, the estimated PEP rate remained significantly higher with FCSEMSs (13.4%) than with PSs (0%; *P* = 0.011). Thus, even after rigorous adjustment for age, sex, stage, tumor location, MPD, and EPS placement, FCSEMS placement was associated with an absolute 13% increased risk of PEP. The four-group comparison according to groove involvement and stent type was intended for descriptive and visual illustration of their interactions rather than as a fully powered subgroup analysis.

**Table:** **Table 5** Baseline characteristics between PS and FC-SEMS groups after IPTW adjustment.

**Variable**	**Category**	**PS**	**FCSEMS**	**SMD**
		**N = 59.5**	**N = 61.7**	
Age		72.64 ± 9.45	72.89 ± 8.81	0.027
Sex	Female	25.9 (43.6)	22.8 (37.0)	0.133
	Male	33.6 (56.4)	38.8 (63.0)	
Clinical stage	IIA	48.6 (81.6)	47.7 (77.3)	0.107
	IIB	10.9 (18.4)	14.0 (22.7)	
Location	Groove	10.6 (17.8)	12.4 (20.0)	0.059
	Head	49.0 (82.2)	49.3 (80.0)	
MPD (< 3 mm)	No	41.0 (68.9)	39.4 (63.9)	0.107
	Yes	18.5 (31.1)	22.3 (36.1)	
EPS placement	No	48.2 (80.9)	50.2 (81.5)	0.014
	Yes	11.4 (19.1)	11.4 (18.5)	
EPS, endoscopic pancreatic duct stent; FCSEMS, fully covered self-expandable metal stent; IPTW, inverse probability of treatment weighting; MPD, main pancreatic duct; PS, plastic stent; SMD, standardized mean difference. Data are shown as mean ± standard deviation for continuous variables and as number (%) for categorical variables. Group comparisons were performed using appropriate statistical tests. Standardized mean differences (SMDs) were calculated to assess covariate balance. Weighted sample sizes are presented as a result of IPTW adjustment.				

## Discussion

This retrospective study addressed two prespecified aims. First, multivariate analysis identified groove involvement as the only pre-procedure anatomical factor independently associated with PEP following placement of an 8-mm FCSEMS. Second, even after IPTW to balance baseline characteristics, incidence of PEP remained significantly higher with FCSEMSs than with PSs (13.4% vs. 0%; *P* = 0.011). These findings suggest that groove involvement, which is readily identifiable on routine cross-sectional imaging, is a practical and objective marker for risk stratification and stent selection in the preoperative setting.

Anatomically, the groove is a narrow area bordered by the pancreatic head, duodenum and the common bile duct. It contains the accessory (Santorini) duct and lies in close proximity to the MPD. Prior studies on groove pancreatitis have shown that fibrosis or inflammation in this area can obstruct the accessory duct and minor papilla, impair collateral drainage, and increase intraductal pressure 16 . Malignant invasion of the groove may compromise both the Santorini duct and MPD. When an FCSEMS is deployed in this setting, the expanded mesh may exert additional external compression on the MPD, potentially elevating ductal pressure and triggering PEP 17 . These pathophysiological mechanisms may account for increased incidence of PEP in patients with groove involvement.

Although most prior risk models for PEP have focused on procedural or pharmacological factors, our study introduced a pre-procedure image-based approach centered on anatomical evaluation of the groove 18 . Although Kozakai et al. and Tamura et al. reported higher PEP rates with FCSEMSs than with PSs in preoperative drainage, neither study accounted for tumor location 19 20 . Umemura et al. proposed that MPD dilatation may protect against PEP by reducing ductal pressure during transpapillary stenting 10 ; however, our data did not identify MPD diameter as a significant modifying factor. In contrast, groove involvement remained a robust anatomical predictor, even after multivariate and IPTW adjustments.

Although a 10-mm FCSEMS is commonly used for biliary drainage, a multicenter randomized trial by Mandai et al. found that FCSEMSs, although requiring fewer reinterventions, were associated with greater intraoperative blood loss, more surgery-related complications, and longer hospital stays than PSs 21 . Based on these findings and discussions with hepatopancreatobiliary surgeons, we prospectively selected an 8-mm FCSEMS to minimize overexpansion and facilitate surgical resection. Our analysis was limited to patients who underwent preoperative biliary drainage, a population in which PEP has a high clinical impact, potentially delaying or precluding NAC or curative surgery. In this context, pre-procedure identification of high-risk anatomical features, such as groove involvement, is of particular relevance. Conversely, unresectable disease is usually managed with larger-diameter metallic stents for palliation at our institution and PSs are rarely selected, which further justified restricting the present analysis to resectable and borderline-resectable cases. This restriction enabled evaluation of PEP risk within a uniform preoperative ERCP strategy, thereby minimizing bias arising from palliative stenting approaches used in unresectable disease.

**Fig. 2:**
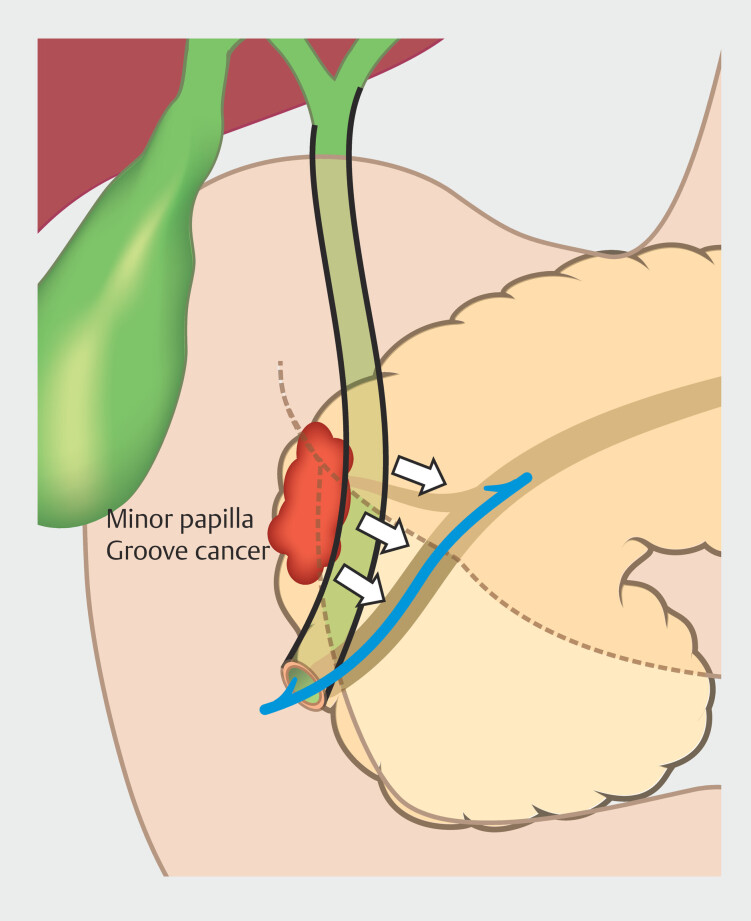
Relationship between groove involvement, minor papilla, and FCSEMS placement.

Prophylactic EPS is generally considered effective in high-risk situations, particularly with FCSEMS placement. Previous studies, including the one by Toyonaga et al. and a recent randomized controlled trial, demonstrated reduced PEP rates when EPS was performed prior to FCSEMS placement. 22 23 . However, routine EPS in all patients may increase procedure complexity and introduce additional risks, particularly in cases with difficult cannulation or a low PEP risk. Our findings suggest that patients with groove involvement may benefit from selective EPS or alternative drainage strategies ( Fig. 2 ).

The present study had some limitations. Although IPTW reduced selection bias by balancing observed covariates, residual confounding by unmeasured variables cannot be excluded. The single-center, retrospective design limits generalizability of the results. Moreover, the number of groove-involved cases was relatively small, which reduced the power of the subgroup analyses. Accordingly, findings from subgroup comparisons should be interpreted as exploratory. Finally, groove involvement was assessed using CT, which may be subject to interobserver variability.

## Conclusions

Within the limitations of the single-center retrospective cohort study, groove involvement was identified as an independent predictor of PEP and use of FCSEMSs was associated with a higher adjusted risk of PEP than PSs. Routine pre-procedure imaging to detect groove involvement may enhance risk stratification prior to biliary drainage. Larger prospective studies are warranted to confirm our findings.

## Supplementary Material

Supplementary Material Zusatzmaterial
