# Supplementary material for: Groove area involvement predicts post-ERCP pancreatitis after 8-mm fully-covered metal stent placement in resectable pancreatic cancer
Source: Endosc Int Open. 2026 Feb 26;14:a28034865. doi: 10.1055/a-2803-4865 (PMC12951030; doi:10.1055/a-2803-4865)
Supplement: Supplementary file 1 — Supplementary Material [file 10-1055-a-2803-4865_28072321.pdf]

Supplementary Table 1 Baseline characteristics in PS and FCSEMS groups before IPTW adjustment.

**Variable**	**Category**	**PS**<br>**n = 17**	**FCSEMS**<br>**n = 45**	**SMD**
Age	1 year	72.00 ± 9.28	73.82 ± 7.90	0.212
Sex	Female	7 (41.2)	16 (35.6)	0.116
	Male	10 (58.8)	29 (64.4)	
Clinical stage	IIA	16 (94.1)	32 (71.1)	0.637
	IIB	1 (5.9)	13 (28.9)	
Location	Groove	4 (23.5)	9 (20.0)	0.086
	Head	13 (76.5)	36 (80.0)	
MPD (< 3 mm)	No	13 (76.5)	30 (66.7)	0.219
	Yes	4 (23.5)	15 (33.3)	
EPS placement	No	10 (58.8)	40 (88.9)	0.728
	Yes	7 (41.2)	5 (11.1)	

EPS, endoscopic pancreatic duct stent; FCSEMS, fully covered self-expandable metal stent; IPTW, inverse probability of treatment weighting; MPD, main pancreatic duct; PS, plastic stent; SMD, standardized mean difference.
Data are shown as mean ± standard deviation for continuous variables and as number (%) for categorical variables. Group comparisons were conducted using appropriate statistical tests. SMDs were calculated to assess covariate imbalance before IPTW adjustment.

Endosc Int Open | DOI 10.1055/a-2803-4865 | © The Author(s).
